# Rapid reverse genetics systems for *Nothobranchius furzeri*, a suitable model organism to study vertebrate aging

**DOI:** 10.1038/s41598-022-15972-3

**Published:** 2022-07-08

**Authors:** Masayuki Oginuma, Moana Nishida, Tomomi Ohmura-Adachi, Kota Abe, Shohei Ogamino, Chihiro Mogi, Hideaki Matsui, Tohru Ishitani

**Affiliations:** 1 Department of Homeostatic Regulation, Division of Cellular and Molecular Biology, Research Institute for Microbial Diseases, Osaka University, Suita, Osaka 565-0871 Japan; 2 Institute for Molecular and Cellular Regulation, Gunma University, Gunma, 371-8512 Japan; 3 Department of Neuroscience of Disease, Brain Research Institute, Niigata University, Niigata, 951-8585 Japan; 4 Center for Infectious Disease Education and Research (CiDER), Osaka University, Suita, Osaka 565-0871 Japan

**Keywords:** Biological techniques, Developmental biology, Genetics, Molecular biology

## Abstract

The African turquoise killifish *Nothobranchius furzeri* (*N. furzeri*) is a useful model organism for studying aging, age-related diseases, and embryonic diapause. CRISPR/Cas9-mediated gene knockout and Tol2 transposon-mediated transgenesis in *N. furzeri* have been reported previously. However, these methods take time to generate knockout and transgenic fish. In addition, knock-in technology that inserts large DNA fragments as fluorescent reporter constructs into the target gene in *N. furzeri* has not yet been established. Here, we show that triple-target CRISPR-mediated single gene disruption efficiently produces whole-body biallelic knockout and enables the examination of gene function in the F0 generation. In addition, we developed a method for creating the knock-in reporter *N. furzeri* without crossing by optimizing the CRISPR/Cas9 system. These methods drastically reduce the duration of experiments, and we think that these advances will accelerate aging and developmental studies using *N. furzeri*.

## Introduction

African turquoise killifish (*N. furzeri*) is an emerging model vertebrate^1,2^. It can be maintained in the laboratory at a low cost, similar to other small laboratory fish, like zebrafish and medaka. Some inbred lines of *N. furzeri* have also been established. Among these, the GRZ strain is the most frequently used line that originates from a sample collected in the Gona‐Re‐Zhou National Park of Zimbabwe^3^. GRZ larvae rapidly grow and mature sexually by 4–5 weeks, with a maximum lifespan of only 3–4 months. This is the shortest life span among vertebrate species and is beneficial for studying aging and age-associated diseases^4^. Furthermore, the developmental process of *N. furzeri* embryo is also quite different from that of zebrafish and medaka embryos and has three unique features; the first one is a slow cell cycle during early cleavage, the second one is the cell dispersion and reaggregation before starting gastrulation, and the third one is the ability to enter diapause, a state of facultative developmental arrest. Diapause allows the organism to survive long-term in extreme environments^5,6^.

To explore gene function, knockout animals and reporter-transgenic animals that enable visualization of gene expression patterns are powerful tools. Methods for CRISPR/Cas9-mediated gene knockout and Tol2 transposon-mediated reporter transgenesis in *N. furzeri* have been previously reported^7,8^. However, these methods require a long time and large breeding areas to produce knockout and transgenic fish. For example, generating the whole-body biallelic knockout of *N. furzeri* by the CRISPR/Cas9 system required at least two rounds of mating that take a further six months, because the body of the first generation-transgenic fish consists of wild-type, heterozygous, and a small percentage of biallelic mutant cells. If the mutant cells contribute to the germline, the next-generation offspring contain hetero-knockout fish, and further crossing can produce homo-knockout fish at a Mendelian rate. Tol2 system-mediated production of fluorescent reporter fish also requires at least one crossing, and it is difficult to judge whether the reporter can reflect endogenous gene expression patterns in the reporter-containing F0 generation. Apart from the Tol2 system, gene knock-in is also useful for generating a reporter *N. furzeri.* Although a gene knock-in technology that inserts 30–50 bp DNA fragments into the *N. furzeri* genome for exchanging a few amino acid sequences has been previously reported^9^, methods for introducing large DNA fragments as fluorescent reporter genes (around 700 bp) have not been established.

In this study, we improved the CRISPR/Cas9 method by introducing three short-guide RNAs (sgRNAs) targeting a single gene to produce a whole-body biallelic knockout in *N. furzeri* in F0 generation with an almost 100% success rate. Furthermore, we created a highly efficient method for generating a fluorescent reporter that reflects endogenous gene expression patterns in F0 generation by injecting Cas9 mRNA with sgRNAs to digest a specific genomic region and donor plasmid containing a heat-shock promoter and fluorescent reporter gene. These methods drastically reduce the time and space required for experiments and we hope that these will accelerate developmental and aging studies using *N. furzeri*.

## Results

### The triple-target CRISPR-mediated efficient production of whole-body biallelic knockout

**Figure 1:**
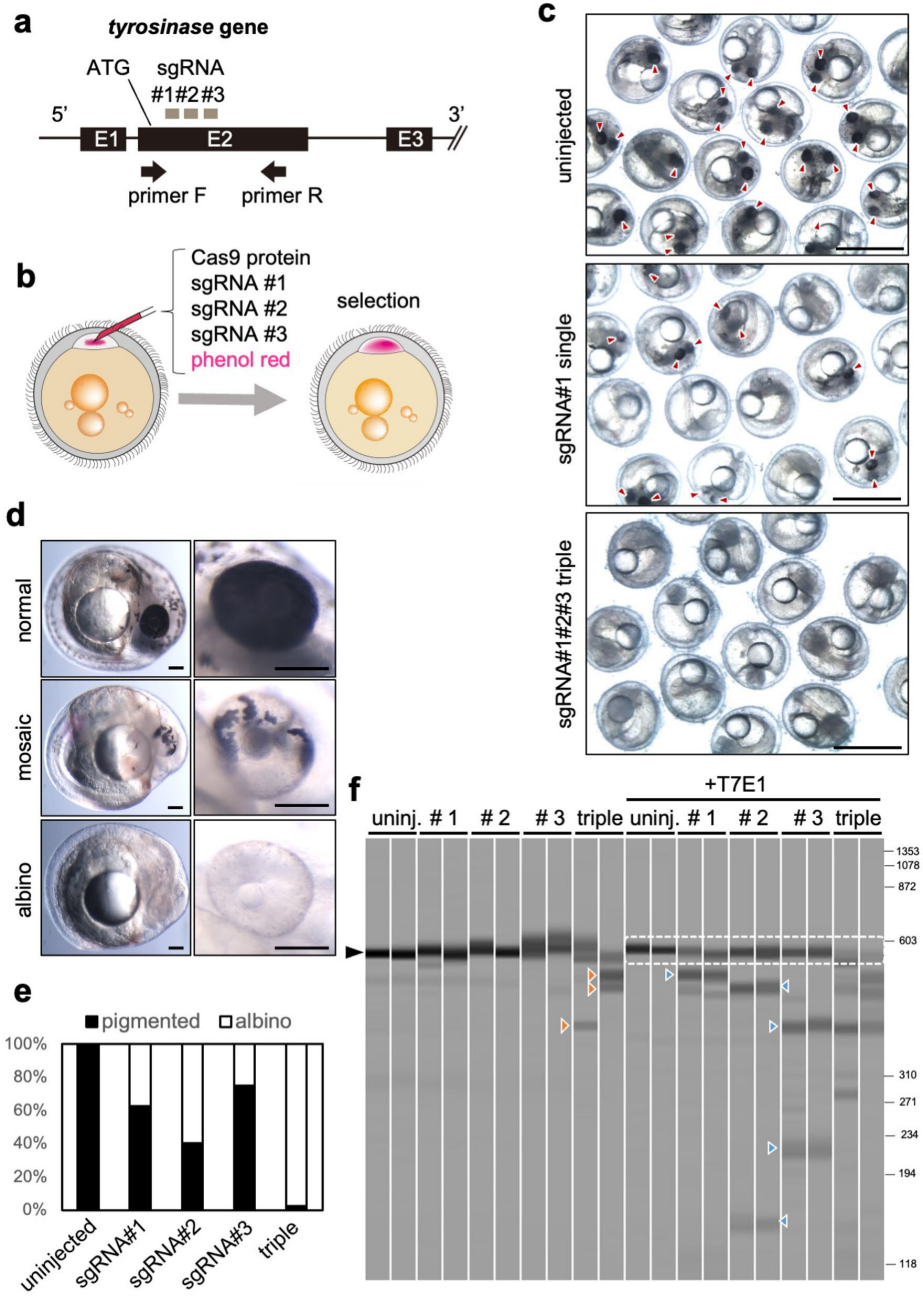
Triple-target CRISPR efficiently produces whole-body biallelic knockout *N. furzeri*. (**a**) A schematic representation of the *tyrosinase* locus (black box: exon, ATG: start codon) and three sgRNA target sites (gray box). Arrow indicate PCR primers used for confirmation of knockout/deletion. (**b**) To make the whole-body biallelic knockout in *N. furzeri*, sgRNA#1–3 for genomic digestion in Exon 2 region, Cas9 protein, and phenol red are co-injected into one-cell-stage embryos. After incubation for one hour, we selected embryos with strong red color in the whole cell body. (**c**) 9 days-post-fertilization (dpf) embryos uninjected or injected with single or triple sgRNAs. Scale bar: 1 mm. Arrowheads indicate pigmented eyes. (**d**) Typical phenotype of sgRNAs-injected embryos at 9 dpf. Left panels show whole bodies and right panels show high magnification of eye regions. Scale bar: 100 μm. (**e**) The percentage of embryos uninjected (n = 37) or injected with single (#1: n = 24, #2: n = 35, #3: n = 20) or triple (n = 41) sgRNAs showing albino or pigmented phenotype at 10 dpf. (**f**) Electrophoresis of PCR products performed using primers described in Fig. 1a with genomic DNA from embryos uninjected or injected with single and triple sgRNA as templates. Each data set was obtained in duplicate. DNA cleavages were detected by the T7E1 assay shown on the right-side lanes. Black arrowheads indicate the expected size of PCR products. Dot line surrounds intact PCR products. Orange and blue arrowheads indicate shortened PCR products, suggesting that the deletion had occurred in the *tyrosinase* gene.

A multiple-target CRISPR strategy, in which multiple sgRNAs are used to target a single gene, has efficiently produced biallelic KO animals in the F0 generation in several model organisms^10,11^. First, to examine whether a multiple-target CRISPR strategy would also work efficiently in *N. furzeri*, we compared the single-and triple-target CRISPR for the rate of generating the whole-body biallelic knockout of the *tyrosinase* gene. The *tyrosinase* gene encodes an enzyme which converts tyrosine into melanin, and loss of *tyrosinase* function results in pigmentation defects. We selected three specific sgRNA target sites on the *tyrosinase* gene that did not overlap with other genomic sequences, were close to the translational initiation sequence using the chop-chop program^12^ (Fig. 1a) and injected sgRNAs individually or in a combination of three with Cas9 protein and phenol red into the cell body of 1-cell stage embryos. After one hour of incubation, the embryos with red-colored cell bodies indicating efficient injection were selected (Fig. 1b). The efficiency of the biallelic disruption of *the tyrosinase* gene was evaluated based on a loss of pigmentation in the eyes, head, and trunk at 9–10 days-post-fertilization (dpf). Injection with single sgRNA induced albino phenotype (complete loss of pigmentation in both eyes, head, and trunk) in about 25–60% of embryos, while the remaining embryos still generated pigmented cells, indicating that they were ‘mosaic’ knockouts (Fig. 1c–e). In contrast, 97.6% of the embryos injected with triple sgRNAs- showed an albino phenotype, suggesting that they were whole body biallelic knockouts (Fig. 1c–e). Consistent with these results, electrophoresis analyses showed that injection of triple sgRNA, but not of single sgRNA, induced deletion of *the tyrosinase* gene (Fig. 1f, in the absence of T7 endonuclease I (T7E1)). In addition, the T7EI assay^13^ revealed that injection of a single sgRNA partially induced a single-site mutation in the *tyrosinase* gene, while the triple sgRNA injection efficiently induced multiple mutations and significantly reduced the intact *tyrosinase* gene (Fig. 1f), suggesting that triple sgRNA produced biallelic mutant cells in the whole body of *N. furzeri* embryos. These results indicate that the triple-target CRISPR can efficiently produce whole-body biallelic knockout in *N. furzeri* in the F0 generation.

**Figure 2:**
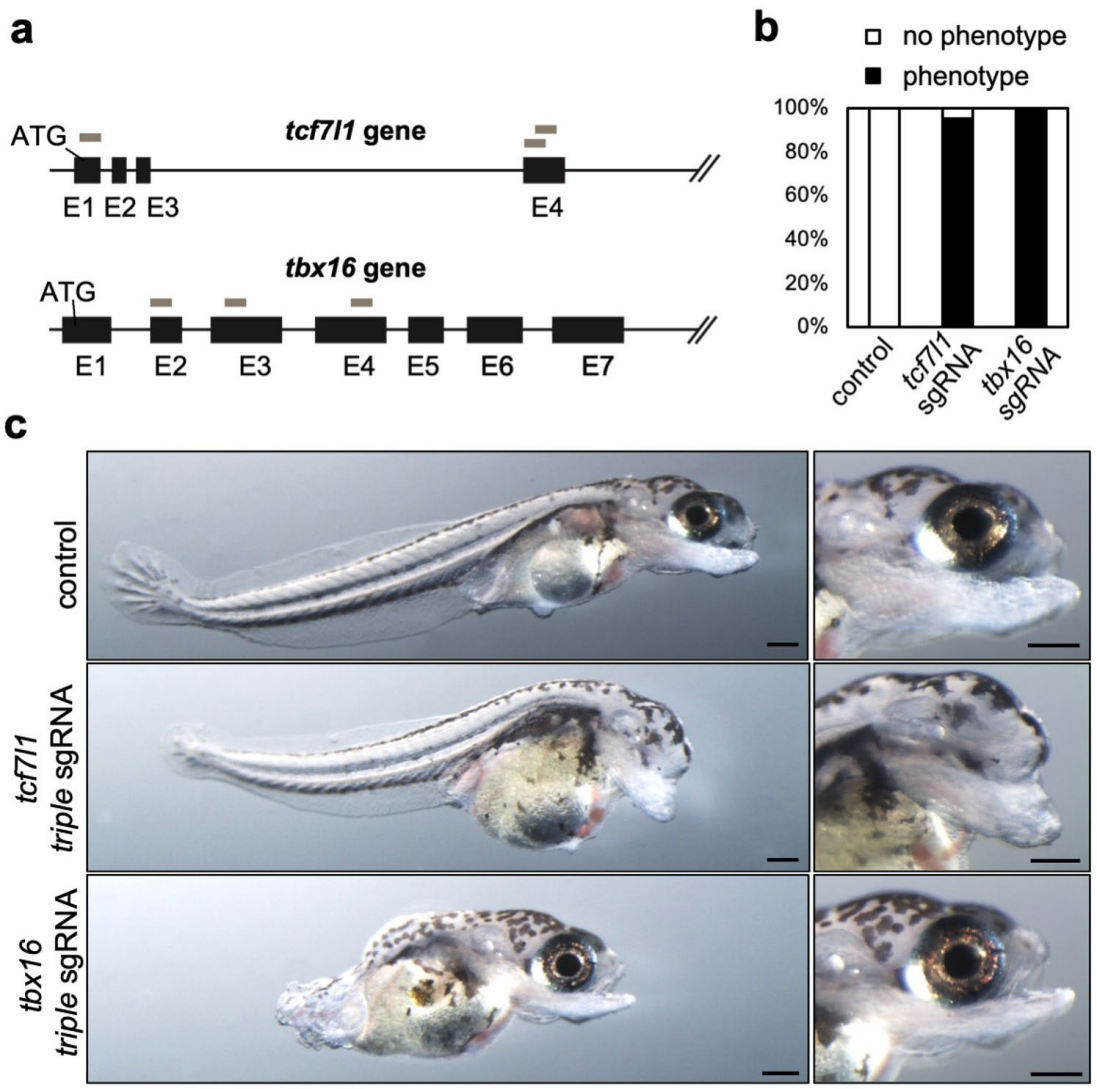
Triple-target CRISPR efficiently disrupts the genes involved in morphogenesis. (**a**) A schematic representation of *tcf7l1* and *tbx16* loci (black box: exon, ATG: start codon) and three sgRNA target sites (gray box). (**b**) The percentage of embryos uninjected (control, n = 63) or injected with triple sgRNA targeting *tcf7l1* (n = 21) or *tbx16* (n = 28), respectively, showing expected phenotype (phenotype) at 14 dpf. (**c**) 14 dpf embryos uninjected (control) or injected with triple sgRNAs targeting *tcf7l1* and *tbx16*, respectively. Scale bar: 100 μm.

Next, to further confirm the efficacy of triple-target CRISPR in *N. furzeri*, we targeted the *tcf7l1* and *tbx16* genes. The phenotypes of these gene knockouts have been well-characterized in zebrafish. The *tcf7l1* gene encodes a transcriptional repressor in the Wnt/β-catenin signaling pathway and zebrafish embryos with *tcf7l1* loss-of-function mutation exhibit a posteriorized phenotype lacking the eye and a part of the forebrain due to excessive activation of Wnt/β-catenin signaling^14^. The *tbx16* gene is expressed in paraxial mesoderm precursors, and loss-of-function of *tbx16* disrupted the paraxial mesoderm tissue from which somites are derived, causing a drastic reduction in the tail region in zebrafish^15^. We designed three specific sgRNAs targeting *tcf7l1* or *tbx16* genomic loci in *N. furzeri* that are close to their translational initiation sequences (Fig. 2a) and injected them in the same way as described above. Remarkably, 95.2% of embryos injected with triple sgRNAs targeting the *tcf7l1b* gene showed a posteriorized phenotype (loss of eyes and reduction of anterior brain regions (Fig. 2b,c), which was similar to that of zebrafish mutants lacking *tcf7l1* gene^14^. Furthermore, 100% of embryos injected with triple sgRNAs targeting the *tbx16* gene exhibited tailless phenotype (Fig. 2b,c), which is the same phenotype observed in zebrafish lacking *tbx16* gene^15^. These results indicate that triple-target CRISPR can be used for several genes, suggesting that this strategy could be applied to systematic functional analysis of genes in *N. furzeri* development and aging models.

### Application of the triple-target CRISPR for mosaic analysis

**Figure 3:**
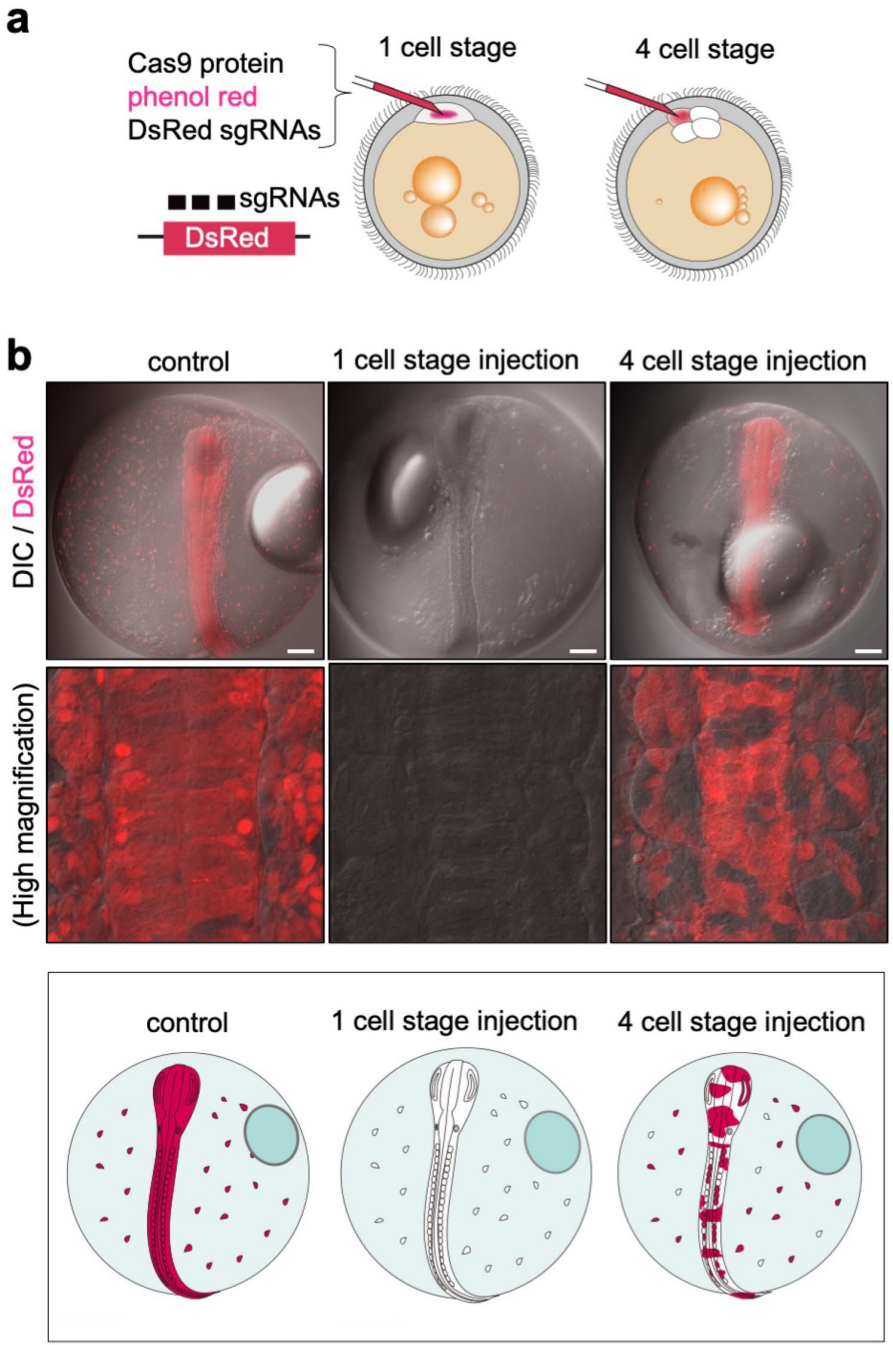
Efficient generation of genetic mosaics in *N. furzeri* embryos. (**a**) Schematic representation of a method for generating whole body and mosaic knockouts. SgRNAs for the DsRed gene, Cas9 protein, and phenol red are co-injected into one-cell-stage or 4-cell-stage embryos. (**b**) DsRed distribution in 7 dpf embryos uninjected (control) or injected with DsRed sgRNAs at 1-cell or 4-cell-stage. The top and middle panels show the whole embryo image and a high magnification image of the trunk region, respectively. Scale bar: 100 μm. The bottom panel shows an illustration of DsRed distribution in the embryos.

Genetic mosaic analysis can be used to gain insights into the cell specificity of gene function. Because the triple-target CRISPR strategy can efficiently and quickly introduce biallelic knockout in the cell receiving the injection and its descendant cells, we believe that this strategy can also be used to stably generate genetic mosaics in *N. furzeri* by injecting gene-targeting triple sgRNAs into a single cell at the multi-cell stage. To test this idea, we established a DsRed2-transgenic *N. furzeri* line, which expresses the red fluorescent protein DsRed2 ubiquitously. Triple sgRNAs targeting the DsRed2 gene were injected into the DsRed2-transgenic eggs (Fig. 3a). In the uninjected control embryos, DsRed2 expression was observed in all cells throughout the body (Fig. 3b). However, the embryos injected with DsRed2-targeting triple sgRNAs into the cell body of 1-cell stage fertilized eggs eliminated DsRed2 expression completely in all the cells, confirming that the triple-target CRISPR produces whole-body knockout (Fig. 3b). Importantly, injection of the triple sgRNAs into the single-cell bodies of the 4-cell stage embryo eliminated DsRed2 expression only in some cells (Fig. 3b). These results indicate that triple-target CRISPR is applicable for generating the genotypically mosaic *N. furzeri*.

### Efficient production of knock-in reporter fish using CRISPR/Cas9

**Figure 4:**
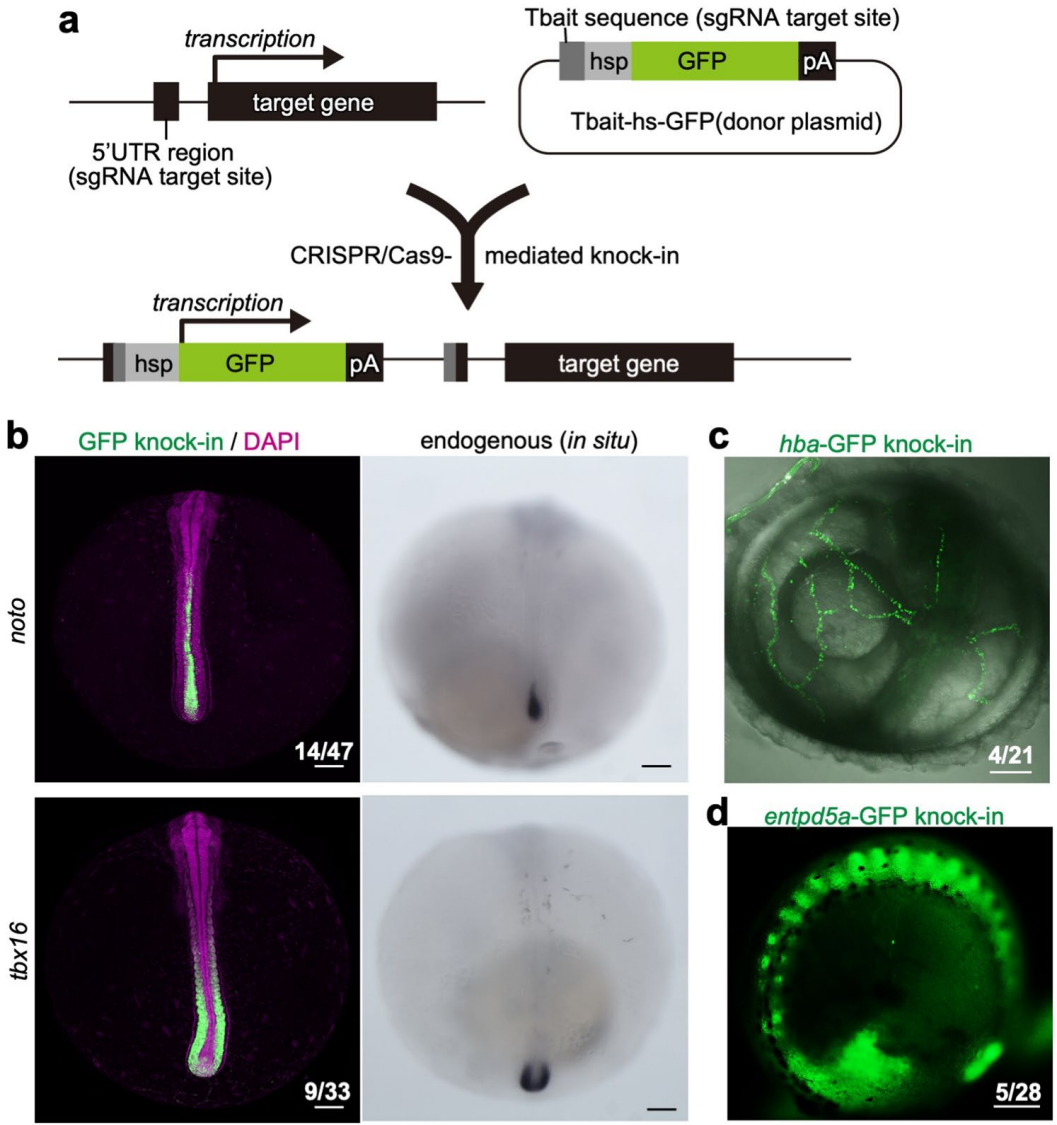
CRISPR/Cas9-mediated generation of reporter knock-in in *N. furzeri*. (**a**) Two sgRNAs for digesting the 5’ UTR region of the target gene and donor plasmid, respectively, were designed for generating knock-in transgenic fish. The donor plasmid with the Tbait sequence and Cas9 mRNA are co-injected with sgRNAs and phenol red into the embryos at the one-cell stage. After injection, CRISPR/Cas9-mediated cleavage occurs at the 5’ UTR region and the Tbait sequence. As a result, causing homology-independent DNA repair to let the donor plasmid knock in into the target locus. The integrations occur in both directions. Cis-regulatory DNA sequence for the target gene expression acts on the *hsp70* promoter (hsp), resulting in the expression of the reporter gene in the cells that express the target gene. pA: polyA. (**b**) The GFP knock-in embryos injected with sgRNA targeting the 5’ UTR region of *noto* (upper panels) or *tbx16* genes (bottom panels). GFP expression reflecting endogenous gene expression patterns was detected in the living embryos injected with sgRNAs targeting *noto* (n = 14/47) and *tbx16* genes (n = 3/9) at 7 dpf (left panels). Expression patterns of endogenous *noto* and *tbx16* were confirmed by in situ hybridization. (**c**,**d**) The GFP knock-in embryos injected with sgRNA targeting the 5’ UTR region of *hba* (**c**) or *entpd5a* genes (**d**). GFP expression reflecting endogenous gene expression patterns was detected in the living embryos sgRNA targeting *hba* (n = 4/21 at 8 dpf) and *entpd5a* genes (n = 5/28 at 28 dpf). See also Supplemental Video 1.

CRISPR/Cas9 was effective in modifying the genome of *N. furzeri,* and we believe that this method is also useful for generating reporter gene knock-in *N. furzeri* at F0 generation. To test this possibility, we applied the protocol for generating the previously reported CRISPR/Cas9-mediated reporter knock-in system in zebrafish or medaka^16,17^. As described in Fig. 4a, this protocol requires two sgRNAs for digesting the target gene on the genome and donor plasmid. One sgRNA targets the 5’-UTR region, approximately 100–200 bp upstream of the translational initiation site of the genomic target gene. The second sgRNA targets the Tbait sequence, upstream of the *hsp70* basal promoter fused with the GFP gene on the donor plasmid. Concurrent digestion of the genome and plasmid DNA by the Cas9 protein results in the integration of the donor plasmid into the genome via a homology-independent repair system. Then, cis-regulatory sequences controlling the spatiotemporal expression pattern of the target gene drive the GFP expression in target gene expressing cells through *the hsp70* promoter (Fig. 4a). We chose *noto* (notochord homeobox) and *tbx16*, the transcripts of which are specifically expressed in the notochord and paraxial mesoderm linage, respectively (Fig. 4b, right), as target genes. Surprisingly, almost 20–30% of embryos strongly and specifically expressed GFP in the area expressing endogenous *noto* or *tbx16* mRNAs (Fig. 4b, left). Using this method, we also succeeded in visualizing the expression of *hemoglobin embryonic subunit alpha* (*hba*) and *ectonucleoside triphosphate diphosphohydrolase 5a* (*entpd5a*) genes, which are well-established markers for erythrocytes and skeletal tissues, respectively, in zebrafish^18,19^ in the F0 generation (Fig. 4c,d, Supplemental Video 1). These results indicate that CRISPR/Cas9-mediated knock-in systems allow for rapid identification of gene expression patterns in *N. furzeri*.

## Discussion

Genetic engineering tools, such as gene knockout and reporter animal models, facilitate the analysis of gene functions and regulation. Many mutant and reporter-transgenic zebrafish lines have been generated and have promoted zebrafish studies, consequently making zebrafish one of the most popular animal models. *N. furzeri* has emerged as a new fish model that allows the study of important biological processes such as aging, age-associated diseases, and embryonic diapause. It is difficult to study these processes using other well-established model organisms, such as mice and zebrafish. Although methods for generating knockout and transgenic lines in *N. furzeri* have been established^7–9^, the number of studies using mutant and transgenic *N. furzeri* is still very limited^5,6,8,9,20–23^. These previously established methods require a long time and large breeding areas to produce knockout and transgenic fish. Therefore, to accelerate *N. furzeri* research, the development of rapid and efficient methods for *N. furzeri* reverse genetics is required. In this study, we successfully developed a method to effectively generate whole-body biallelic knockout *N. furzeri* without crossing. We also established a method for monitoring endogenous gene expression patterns by creating a knock-in reporter gene without crossing. These methods for *N. furzeri* without the need for crossing would facilitate not only the identification of genes involved in vertebrate aging and diapause but also an understanding of their expression patterns. Furthermore, we established a method to generate genetically mosaic organisms. This method enables us to understand the molecular mechanism in more detail, allowing us to examine the non-autonomous functions of the genes related to aging and diapause, such as cell–cell and/or organ-organ interaction processes. These sequential analyses in *N. furzeri* may facilitate rapid identification of the genes and pathways that are critical for aging and diapause throwing light on the unexplored biological phenomena.

## Methods

### Ethical approval

All experimental animal care was performed in accordance with institutional and national guidelines and regulations. The study protocol was approved by the Institutional Animal Care and Use Committee of the respective universities (Gunma University Permit# 17–051; Osaka University, RIMD Permit# R02-04). The study is reported in accordance with ARRIVE guidelines.

### Maintenance of fish lines

The GRZ (GRZ-AD) strain of *N. furzeri*^3^ was gifted by Prof. Adam Antebi. Fish were maintained at 26.5 °C, 0.7 conductivity on 12/12 h light/dark cycle in the fish breeding system (MEITO, Nagoya, Japan) at a density of 1 fish per 1.4 L tank for adult fish. Fish were fed freshly hatched brine shrimp twice a day from Monday to Saturday, and once a day on Sunday, fish that were over 2 weeks old were also fed bloodworms (Kyorin, Himeji, Japan). For mating, one adult male and 3–4 female fish were kept in 4 L tanks, and females spawned on a sand substrate in the plastic cases. Embryos were collected and incubated in the egg water (0.03% sea salt containing methylene blue). Ten days later, the embryos were plated on sterile dry peat moss until they were ready to hatch. Usually, one month after egg collection, embryos were ready to hatch and incubated in the 0.07% ice-cold humic acid solution (53680, Sigma-Aldrich, St. Louis, MO, USA) for 30–60 min, and transferred into the 4 L hatching tank with air supply. Hatched fry were kept in a hatching tank, and half of the solution was changed with the fish breeding system water every day. After 2 weeks, by which time they had grown to be around 1–1.5 cm, they were transferred to 1.4 L tanks similar to the adult fish.

### Triple-target CRISPR

The sgRNA targeting sites are listed in Supplementary table 1 and the gene sequences were retrieved using NCBI Gene IDs (*tyrosinase*:107380455, *tcf7l1*:107393717, *tbx16*:107393822). We selected three target sites on the protein coding sequences for each gene that did not overlap with other genomic sequences and were close to the translational initiation sequence, aiming to induce frameshift mutation, using the chop-chop program. The protocol for sgRNA synthesis was based on a previously reported method^24^. The oligonucleotides containing a T7 promoter sequence, target sequence, and the sgRNA templates were PCR-amplified from pDR274 vectors^25^ using the oligonucleotides and primer sgRNA-RV with PrimeSTAR Max (TaKaRa, Kusatsu, Japan) and purified using the NucleoSpin Gel and PCR Clean-up Kit (MACHEREY–NAGEL, Düren, Germany). sgRNAs were synthesized using the CUGA7 gRNA Synthesis Kit (Nippon Gene, Tokyo, Japan), and their concentrations were measured using a NanoDrop Lite spectrophotometer (Thermo Fisher Scientific, Waltham, MA, USA). The injection solutions consisted of three sgRNAs (20 pg each), Cas9 protein (1 nM, M0646, New England Biolabs, MA, USA), and phenol red (P0290, Sigma-Aldrich) in RNase-free water. The solution was injected into the cell body of 1-cell stage fertilized eggs or 4-cells stage embryos to generate genetic mosaics, and then the embryos with the red-colored cell body were selected. The DsRed2-transgenic line was created by injecting the plasmid (Ola.Actb:LOXP-DsRed2-LOXP-EGFP)^26^ with 15 pg *tol2* mRNA. The efficiency of DNA digestion was analyzed by PCR, including the targeting site following the primer (listed in Supplementary table 1) and T7E1 reaction (313-08801, NIPPONGENE), and then PCR fragments were analyzed by a microchip electrophoresis system (MCE-202 MultiNA, Shimadzu, Kyoto, Japan) according to the manufacturer's instructions using a DNA-1000 reagent kit (Shimadzu).

### CRISPR/Cas9-mediated reporter knock-in

A donor DNA plasmid (pBS-Tbait-olhs-GFP) and sgRNA for the bait sequence were prepared according to a previously described method^15,16^. Capped mRNA encoding Cas9 nuclease was synthesized using the pCS2 + hSpCas9 vector as a template using the mMessage mMachine SP6 Kit (Thermo Fisher Scientific). Targeting sites of sgRNA (listed in Supplementary table 1) were selected from the 5’ UTR region using the chop-chop program, and genes sequences were retrieved from the NCBI Gene database (Gene ID of *noto*:107391787, *tbx16*:107393822, *hba*:107378369, *entpd5a*:107392375).

### Microinjection

The injection plates were generated by melting 2% agarose with egg water in a 100 mm petri-dish, and the injection mold was floated with 0.9 mm width 1.0 mm height (AM6540-0904-1, IPN-Factory, Hekinan, Japan). For injection needle preparation, a glass capillary (G-1, Narishige, Tokyo, Japan) was pulled by one-step pulling using a PC-10 puller (Narishige), and the tip of the capillary was slightly broken off. RNA and DNA solutions were introduced into the needle using a microloader (5242956003, Eppendorf) and injected using an IM 300 microinjector (Narishige).

### In situ hybridization

Embryos were fixed with 4% paraformaldehyde. Whole-mount in situ hybridization was performed according to a standard protocol. Probes for the *noto* and *tbx16* genes were generated from *N. furzeri* embryonic cDNA by PCR using the sequences retrieved using the gene IDs from the NCBI Gene database (*noto*:107391787, *tbx16:*107393822).

### Imaging

Bright-field images of embryos and fry were captured using a stereomicroscope (Leica, Wetzlar, Germany). Fluorescent images of the embryos were captured using FV3000 (Olympus, Tokyo, Japan).

## Supplementary Information

Supplementary Information 1.

Supplementary Legends.

Supplementary Video 1.

## Data Availability

The datasets analyzed during the current study are available in the NCBI repository, [*tyrosinase*:107380455, *tcf7l1*:107393717, *tbx16*:107393822, *noto*:107391787, *hba*:107378369, *entpd5a*:107392375].
