# Supplementary material for: Rapid reverse genetics systems for Nothobranchius furzeri, a suitable model organism to study vertebrate aging
Source: Sci Rep. 2022 Jul 8;12:11628. doi: 10.1038/s41598-022-15972-3 (PMC9270483; doi:10.1038/s41598-022-15972-3)
Supplement: Supplementary file 1 — Supplementary Information 1. [file 41598_2022_15972_MOESM1_ESM.pdf]

Supplemental Table 1

sgRNA for Triple CRISPR

Target_gene	Target_sequence
tyrosinase#1	CGGCCTTGTGCCAACTCAGAGGG
tyrosinase#2	GAACCGTCTCCCTCCCACACCGG
tyrosinase#3	CAGGTCTCAGACGAGCCCAATGG
tbx16#1	GATAGAATCACTTTACAGTGTGG
tbx16#2	TGGGTGGAGGTAGGTCCGGCAGG
tbx16#3	ACCGCGATGAAGGAAGTCTCTGG
tcf7l1#1	CGAAAACGTCTCCGCGGAGAGGG
tcf7l1#2	ACGGGAGTCTTTGATTCCTGCGG
tcf7l1#3	GACTCCCGTTCTCCAACACCAGG
DsRed2#1	GGCCACGAGTTCGAGATCGAGGG
DsRed2#2	CTCGGTGATGACGTTCTCGGAGG
DsRed2#3	GGAGCCGTACTGGAACTGGGGGG

sgRNA for Knock-in

Target_gene	Target_sequence
noto	CGCGGTTGTTCCTACTAGGCAGG
tbx16	AGGGTTCCCCTTAAAAGTCATGG
hba	TAAGTCTTCAGGATGTAGCTGGG
entpd5a	CTTAGTTCAGCTCACAGGAGGG
tbait	GGCTGCTGTCAGGGAGCTCATGG

Genotyping

gene_name	Primer_frd	Primer_Rv
tyrosinase	ATGAGGAGCCTGCTTGTGTC	CCTCCTAGGAAGGCGTCTCT

Probe

gene_name	Primer_frd	Primer_Rv
tbx16	ATCATTCTGCACTCCATGCATCGCTA	GTAGTCAGCAGTCAGAGGGTGAG
noto	TTAACAGGGCTCTGGAGAGTCGTC	ATGCAGGTACCGAACACACCGATGGG
