# Supplementary material for: Rapid reverse genetics systems for Nothobranchius furzeri, a suitable model organism to study vertebrate aging
Source: Sci Rep. 2022 Jul 8;12:11628. doi: 10.1038/s41598-022-15972-3 (PMC9270483; doi:10.1038/s41598-022-15972-3)
Supplement: Supplementary file 2 — Supplementary Legends. [file 41598_2022_15972_MOESM2_ESM.docx]

Supplemental Video 1: **Time-lapse imaging of *hba* reporter expression in the GFP knock-in *N. furzeri* embryos.** See also Fig 4c.
